# Applicability of Artificial Vascularized Liver Tissue to Proteomic Analysis

**DOI:** 10.3390/mi12040418

**Published:** 2021-04-11

**Authors:** Nobuhito Mori, Yasuyuki S. Kida

**Affiliations:** 1 Cellular and Molecular Biotechnology Research Institute, National Institute of Advanced Industrial Science and Technology (AIST), Central 5-41, 1-1-1 Higashi, Tsukuba, Ibaraki 305-8565, Japan; n-mori@aist.go.jp; 2 Advanced Photonics and Biosensing Open Innovation Laboratory, AIST, Central 5-41, 1-1-1 Higashi, Tsukuba, Ibaraki 305-8565, Japan

**Keywords:** blood vessels, hepatocellular carcinoma, liver, organoids, proteome

## Abstract

Artificial vascularized tubular liver tissue has perfusable blood vessels that allow fluid access to the tissue interior, enabling the injection of drugs and collection of metabolites, which are valuable for drug discovery. It is amenable to standard evaluation methods, such as paraffin-embedded sectioning, qPCR, and RNA sequencing, which makes it easy to implement into existing research processes. However, the application of tissues vascularized by the self-assembly of cells, (including tubular liver tissue, has not yet been tested in comprehensive proteomic analysis relevant for drug discovery. Here, we established a method to efficiently separate cells from the tubular liver tissue by adding a pipetting step during collagenase treatment. By using this method, we succeeded in obtaining a sufficient number of cells for the proteomic analysis. In addition, to validate this approach, we compared the cells separated from the tissue with those grown in 2D culture, focusing on the proteins related to drug metabolism. We found that the levels of proteins involved in metabolic phases II and III were slightly higher in the tubular liver tissue than those in the 2D cell culture. Taken together, our suggested method demonstrates the applicability of tubular liver tissue to the proteomic analysis in drug assays.

## 1. Introduction

Artificial liver tissue, in which hepatic parenchymal and non-parenchymal cells are combined and cultured with scaffold materials in three dimensions, has been actively developed as a tool for evaluating drug efficacy and hepatotoxicity in the field of drug discovery. This can be attributed to the fact that, unlike two-dimensional culture systems, it has a structure and function similar to that of the living liver [1,2,3,4,5,6,7]. Recently, we developed a tubular liver tissue with both main perfusable blood vessels and a capillary network formed by the self-assembly of cells [8,9]. The tissue was engineered by using a perfusion device that interfaces with a fluid delivery system to solve the problem of the lack of perfusable blood vessels in conventional liver tissue. The perfusable blood vessels of the tubular liver tissue enable fluidic access to the interior of the tissue, which was difficult previously. Therefore, it is now possible to maintain cell density and liver function in the tissue by perfusing culture medium to collect albumin produced in the tissue, or to measure metabolism by injecting drugs. In addition, the tubular liver tissue has sufficient size and cell density to enable the application of various methods commonly used in the evaluation of biological tissues, such as paraffin-embedded sectioning, qPCR, and RNA sequencing. Thus, the tubular liver tissue is highly compatible with existing molecular and cellular techniques.

However, the applicability of the proteome analysis methods [10] to tissues which are densely compacted and vascularized by the self-assembly of cells, such as the tubular liver tissue, has not been verified. As tissue function and drug effects depend, to a large extent, on proteins, proteomic analysis, which is a comprehensive quantitative technique to study protein composition, is an important evaluation method in drug discovery [11,12]. For example, it has been reported that the transcriptome and proteome do not always coincide [13,14], and thus it is necessary to examine both when investigating the effects and mechanisms of action of drugs. In addition, proteomic analysis is powerful in cases where phosphorylation plays an important role in the drug mechanism [15,16]. In this study, we formulated a method to construct tubular liver tissue by using a perfusion culture device, and performed proteomic analysis by data-independent acquisition (DIA) mass spectrometry (MS). Furthermore, to validate this approach we compared the proteome of the tubular liver tissue consisting of HepG2 cells, human umbilical vein endothelial cells (HUVECs), mesenchymal stem cells (MSCs), and collagen gels, with that of cells in two-dimensional (2D) culture. In addition to the perspective analysis of the entire obtained dataset, we focused on the proteins involved in phase I (oxidation; reduction, hydroxylation, hydrolysis, etc.), phase II (glutathione conjugation, glucuronide conjugation, sulfate conjugation, etc.), and phase III (transport) of drug metabolism while comparing proteomes, as these proteins are considered particularly important in drug discovery. The developed method for proteomic analysis of tubular liver tissue will be useful when performing drug assays.

## 2. Materials and Methods

### 2.1. Two-Dimensional Cell Culture

To construct the tubular liver tissue, HepG2 cells, HUVECs, and MSCs were expanded in culture dishes. HepG2 cells (JCRB1054, Japanese Collection of Research Bioresources Cell Bank, Osaka, Japan) were cultured in low glucose Dulbecco’s modified Eagle’s medium (DMEM) (FUJIFILM Wako Pure Chemical Corporation, Osaka, Japan) with 10% fetal bovine serum (FBS) (Biosera, Kansas City, MO, USA) and 1% penicillin-streptomycin solution (PS) (FUJIFILM Wako Pure Chemical). HUVECs (PromoCell GmbH, Heidelberg, Germany) were cultured in endothelial cell growth medium 2 (EGM2) (PromoCell), with a supplement kit and 1% PS. MSCs (SCRC-4000, ATCC, Manassas, VA, USA) were cultured in high-glucose DMEM, supplemented with 10% FBS, 1% non-essential amino acids solution (FUJIFILM Wako Pure Chemical), and 1% PS. In 2D cultures, all cell types were cultured independently in a mixture of equal volumes of the media for HepG2 cells and HUVECs in order to make conditions equivalent to those of the tubular liver tissue.

### 2.2. Preparation of the Tubular Liver Tissue

**Figure 1:**
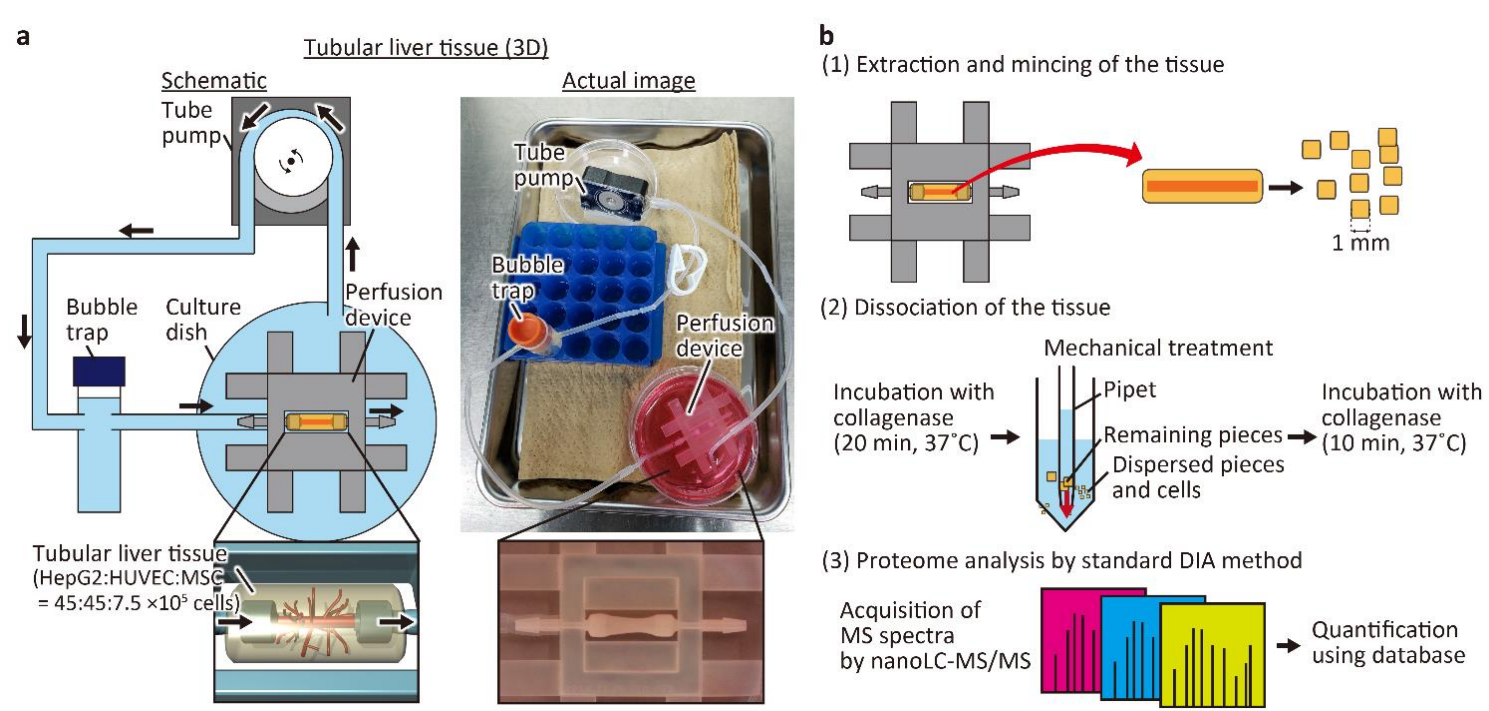
Conceptual diagram and procedure of the experiment. (**a**) Schematic and actual images of the perfusion system for the tubular liver tissue. The medium in the culture dish is aspirated by the tube pump, passed through the bubble trap, and delivered to the tubular liver tissue in the perfusion device. The medium that has passed through the tubular liver tissue is released into the culture dish and circulated again by the tube pump. (**b**) Proteomic analysis procedure of the tubular liver tissue.

The tubular liver tissue was constructed according to the previously a reported method (Figure 1a) [8]. Briefly, a perfusion device was first fabricated using a 3D printer, hydrophilized by air plasma, and then the inside of the device was coated with fibronectin. After inserting a 25 G needle between the connectors, 150 μL of neutralized collagen solution (IAC-50, Koken Co., Tokyo, Japan) with suspended HepG2 cells (3 × 10^7^ cells/mL), HUVECs (3 × 10^7^ cells/mL), and MSCs (0.5 × 10^7^ cells/mL) was injected into the device to construct the tissue. Then, the needle was removed to form a hollow channel inside the tissue, and HUVECs were seeded inside the channel to form the main blood vessel. Finally, the tissue was connected to a tube pump (RP-HX01S, Aquatech Co., Ltd.; Osaka, Japan) and perfused with the medium at a flow rate of 1 mL/h for 6 days. The culture medium was a mixture of equal parts of EGM2 and DMEM.

### 2.3. Histological Analysis

To confirm the quality of the prepared tubular liver tissue, paraffin-embedded tissue sections were prepared for observation. The tissues were fixed with 4% paraformaldehyde (FUJIFILM Wako Pure Chemical) for 6 h at 4 °C, dehydrated with ethanol, permeabilized with Lemosol (FUJIFILM Wako Pure Chemical), and embedded in paraffin. Then, sections of 5 μm thickness were prepared. After deparaffinization by Lemosol and ethanol, the sections were subjected to staining with hematoxylin and eosin (HE) and immunostaining. For immunohistochemistry (IHC), after heat-induced epitope retrieval (HIER) using citrate buffer (pH 6), the sections were incubated with the primary antibody for CD31 (1:100, ab28364, Abcam PLC, Cambridge, UK), followed by a secondary antibody (ImmPRESS Reagent, Anti-Rabbit Ig, Vector Laboratories, Inc., Burlingame, CA, USA). Finally, the sections were colorized with DAB (ImmPACT DAB, Vector Laboratories) and counterstained with hematoxylin. For immunofluorescence, after HIER, the sections were incubated with the primary antibodies against CD31 and CK18 (1:200, MA5-12104, Thermo Fisher Scientific Inc., Waltham, MA, USA) and secondary antibodies (1:200, Alexa Fluor 488-conjugated anti-mouse and Alexa Fluor 555-conjugated anti-rabbit, Thermo Fisher Scientific Inc.).

### 2.4. Cell Collection and Proteomic Analysis

To perform proteomic analysis of the tubular liver tissue, collagen was removed and the cells were collected as shown in Figure 1b. First, the constructed tissue was removed from the device with tweezers and then the tissue was minced with a scalpel to ~1 mm^2^ square pieces. Then, the minced tissue pieces were placed in 10 mL of DMEM containing 2 mg/mL collagenase type I (031-17601, FUJIFILM Wako Pure Chemical) and incubated at 37 °C for 20 min with frequent and mild agitation. Tissue pieces that were not fully digested by collagenase were mechanically dispersed by pipetting several times, and incubated for another 10 min. The cell suspension was filtered through a cell strainer with a pore size of 100 μm and then centrifuged at 200× *g* for 5 min with 20 mL of phosphate-buffered saline (PBS). The cells were suspended in 30 mL of PBS, and collected by centrifugation twice. The cells were stored at −80 °C until they were used for proteomic analysis. In 2D cultured cells used for comparison with the tubular liver tissue, HepG2 cells, HUVECs, and MSCs were independently cultured in a mixture of DMEM and EGM2 in equal amounts, detached by TrypLE Express (Thermo Fisher Scientific Inc.), collected by centrifugation, suspended in PBS, and mixed in the same ratio as in the tubular liver tissue. The cells were washed twice with PBS and then stored at −80 °C.

DIA proteomic analysis was performed by Kazusa Genome Technologies, Inc. according to the previously reported method [17]. In brief, the preprocessing stage involved protein extraction and peptide fragmentation by trypsin. Subsequently, MS measurements were performed by nano liquid chromatography with tandem MS (UltiMate 3000 RSLCnano LC System, Q Exactive HF-X, Thermo Fisher Scientific Inc.). From the acquired MS data, peptides and proteins with both peptide and protein false discovery rate (FDR) below 1% were identified and quantified using Scaffold DIA (Proteome Software, Inc., Portland, OR, USA).

Protein quantification data obtained via DIA proteomic analysis were analyzed and plotted using Microsoft Excel. The Gene Ontology (GO) enrichment analysis was performed by DAVID [18] using RDAVIDWebService package [19] in R [20]. The GO term category was GO_BP_DIRECT.

## 3. Results and Discussion

### 3.1. Evaluation of Tissue Morphology

**Figure 2:**
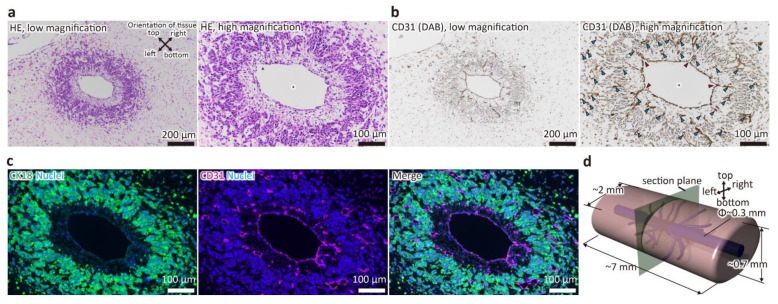
Stained image of a cross-section of the tubular liver tissue perpendicular to the main blood vessel. (**a**) hematoxylin and eosin (HE) staining image. (**b**) immunohistochemistry (IHC) image (CD31). Asterisks indicate the main blood vessels. Blue arrowheads indicate capillaries. Red arrowheads indicate branches from main vessels to capillaries. (**c**) Immunofluorescence image (CK18 and CD31). (**d**) Schematic image of the tubular liver tissue.

To confirm that the samples were properly constructed and that the liver tissue had main blood vessels and capillary network as previously reported, H&E staining of FFPE sections and immunostaining for CD31, a marker for HUVECs, were performed (Figure 2). As shown in Figure 2a, the main blood vessel with a diameter of about 300 μm was maintained, and a region of densely aggregated cells was observed around it. In addition, this area was organized within approximately 200–300 μm from the wall of the main vessel. This indicates that the main vessel was maintained by the hydrostatic pressure of the fluid, and that oxygen and nutrients were supplied to the surrounding space from the medium that flowed through the main vessel as a result of the perfusion, as designed. Furthermore, as shown in Figure 2b,c, the wall of the main vessel was positive for CD31, indicating that it was covered by HUVECs. In addition, CD31-positive luminal structures forming the capillary network were present in the surrounding cell aggregation area, which was primarily composed of CK18-positive cells (i.e., HepG2 cells). Additionally, branches from the main blood vessel to the capillary network were observed. Interestingly, between the main vessel and the cell aggregation area, an area composed of extracellular matrices and a small number of cells negative for both CD31 and CK18, which were presumed to be MSCs, was formed. This structure is similar to the adventitia of real blood vessels. These results indicated that the liver tissue with main blood vessels and capillary network was constructed normally, and thus, the subsequent proteomic analysis was carried out.

### 3.2. Evaluation of the Proteomic Analysis Process

First, to determine whether sufficient tissue was available to obtain the number of cells required for the proteomic analysis, we performed enzymatic dissociation, mechanical dispersion, another round of enzymatic treatment, and collection of the liver tissue cells (Figure 1b). In total, (5.25 ± 0.39) × 10^5^ cells/device were obtained (Table 1). We also tried the method of only enzymatic treatment for 30 min, without dispersion of the tissue fragments by pipetting, but the number of cells obtained was almost 20-fold lower (2.64 × 10^4^ cells/device). As cells and collagen in the tubular liver tissue are tightly packed because of cellular self-assembly, mechanical dispersion had to be performed during the enzymatic treatment for better cell yield. As the number of cells required for the identification and relative quantification of the expressed proteins in DIA proteomic analysis is 5 × 10^5^ cells, the analysis could be performed using only one or two devices. Furthermore, the knowledge of the number of cells that could be obtained from our device in this study is useful because the required number of cells may change, depending on the device used and proteomic analysis method employed.

Next, we performed proteomic analysis of the cells from the tubular liver tissue and 2D culture in the quantities indicated in Table 1. As a result, we successfully identified and quantified 6032 and 6034 proteins from tubular liver tissue and 2D cell culture, respectively. Since the conditions employed for this analysis were such that 3000–5000 proteins could normally be identified and quantified, and the proteomic analysis of 2D cultures is a well-established method [17], it can be concluded that detailed proteomic analysis can be performed using tubular liver tissue samples.

### 3.3. Proteomic Comparison between the Tubular Liver Tissue and 2D Culture

**Figure 3:**
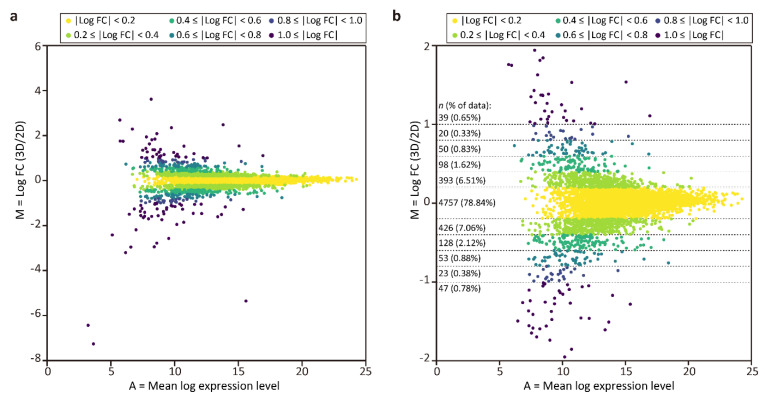
MA plots of proteomic analysis results. (**a**) Plot of all quantified proteins. (**b**) Magnified view of the plot (**a**) in the range of |log_2_ FC| ≤ 2.

To compare protein expression in the tubular liver tissue and 2D culture, MA plots were generated (Figure 3), where M = log_2_ (fold change (FC)) = log_2_ (expression level in tubular liver tissue) − log_2_ (expression level in 2D culture), and A = (log_2_ (expression level in tubular liver tissue) + log_2_ (expression level in 2D culture))/2. This graph shows that the frequencies of log_2_ FC values were unimodal and symmetrically distributed, with the top being 4757 (78.84%) in the range of|log_2_ FC| < 0.2, and that the majority (98.57%) were distributed in the range of|log_2_ FC| < 1.0. Next, we performed GO enrichment analysis among the proteins with log_2_ FC ≥ 0.4 (top 3.43%) and log_2_ FC ≤ −0.4 (bottom 4.16%) to determine the differences between the tubular liver tissue and 2D culture. We found that the GO terms enriched in the tubular liver tissue included peptidyl-serine phosphorylation, glycolytic process, and dephosphorylation (Table 2), suggesting that the cells were more active in the steady state than in 2D culture. The GO terms enriched in 2D culture cells included focal adhesion assembly, positive regulation of cell growth; mitochondrion morphogenesis, mitotic spindle assembly, and checkpoint (Table 3). The relative abundance of protein signals associated with cell adhesion and proliferation could be attributed to the fact that 2D culture cells were cultured on a hard plastic surface, unlike the tubular liver tissue cells.

**Figure 4:**
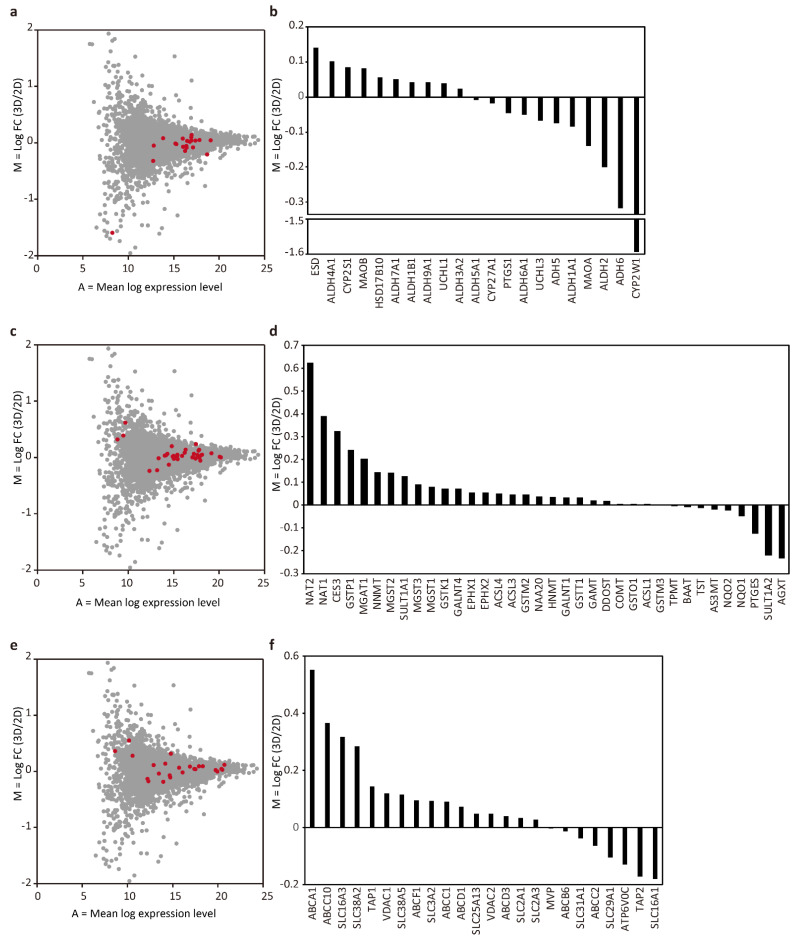
MA plot and bar graph showing log_2_ FC of phase I (**a**,**b**), II (**c**,**d**), and III (**e**,**f**) proteins.

Next, we focused our analysis on the proteins involved in drug metabolism. The protein groups involved in metabolic phases I, II, and III were selected based on the previous reports [9,21]. Among phase I proteins, comparison of the tubular liver tissue and 2D culture data revealed 10 proteins with log_2_ FC > 0, and 11 proteins with log_2_ FC < 0 which can be considered as neutral results (Figure 4a,b). However, it is interesting to note that CYP2W1 was highly expressed in 2D cultures (log_2_ FC = −1.59). Given that CYP2W1 is mainly expressed in tumors [22], 2D cultures resemble tumors in this respect, whereas the lower level of CYP2W1 in the tubular liver tissue makes them similar to normal cells.

There were 27 proteins with log_2_ FC > 0 and 9 proteins with log_2_ FC < 0 among phase II proteins, indicating that many of those proteins, NAT2 in particular (log_2_ FC = 0.62), were highly expressed in the tubular liver tissue (Figure 4c,d). NAT2 is an important protein involved in the metabolism of clinically important drugs such as the anti-tuberculosis drug isoniazid, the anti-epileptic drug clonazepam, the anti-arrhythmic drug procainamide, the sleeping pill nitrazepam, the antihypertensive drug hydralazine, and the antibacterial drug sulfamethoxazole.

Many phase III proteins were also highly expressed in the tubular liver tissue: 16 with log_2_ FC > 0 and 8 with log_2_ FC < 0 (Figure 4e,f). Among the proteins with high expression levels, ABCC10 (log FC = 0.37) is an important protein involved in multidrug resistance because the tubular liver tissue used in this study was constructed using the cancer cell line HepG2, which could be used as tumor tissue for the evaluation of anticancer drugs. Taken together, we found that the expression of many proteins related to drug metabolism, especially in phase II and III, tended to be slightly upregulated in the tubular liver tissue as compared to 2D cultures. This may be an advantage if the tubular liver tissue were to be used for drug assays. In this analysis, the initial cell ratio of the tissue was used as the basis for comparison with 2D culture, in accordance with previous studies [9,23]. However, a more accurate comparison might be performed based on the cell ratio of the tissue after culture, which can be measured by cell sorting. In addition, although we cultured the tissues for 6 days, which was considered sufficient to identify the differences between the 2D and 3D culture according to a previous study [7], it is presumed that a clearer difference in proteome can be detected by extending the culture period. Furthermore, because this study used HepG2, a cancer-derived cell line, there was a limitation in extrapolating the measurement results to normal tissues in drug assays. In the future, it is expected to be possible to construct artificial liver tissues that are similar to normal tissues by using primary hepatocytes, the ‘gold standard’ in drug discovery, or, iPS cell-derived hepatocytes, which have been reported to have a protein expression profile similar to that of primary hepatocytes (compared to the 3D spheroid culture of HepG2) [24]. To construct such tissues, it would be important to select an appropriate medium that can support all cell types, as well as to identify the perfusion conditions that can maximize the hepatocyte support provided by vascular endothelial cells [25]. The insights obtained in this study will be useful when such tissues are established and used for drug assays.

## 4. Conclusions

In this study, we have validated a method for proteomic analysis of tubular liver tissue, which is an artificial liver tissue with blood vessels, constructed using a perfusion device. By removing the tubular liver tissue from the device, and separating the cells from the collagen by collagenase treatment and mechanical dispersion, we succeeded in collecting enough cells for DIA proteomic analysis. Furthermore, by using the obtained cells for proteomic analysis in accordance with existing methods, we identified and quantified proteins, and compared them to samples from 2D cultures. Furthermore, we compared the expression levels of proteins associated with drug metabolism in the tubular liver tissue with that in 2D culture, and found that the expression of proteins involved in metabolic phases II and III tended to be higher in the tubular liver tissue. Taken together, the method developed in this study allows for proteomic analysis of tubular liver tissue and other densely compacted tissues vascularized by the self-assembly of different cells in drug assays.

## Figures and Tables

**Table 1:** Quantitative characteristics of the experimental systems and proteomic analysis output.

Culture Method	Number of Obtainable Cells	Number of Cells Used for Proteomic Analysis	Number of Identified and Quantified Proteins
Tubular liver tissue	(5.25 ± 0.39) × 10^5^/device	8.62 × 10^5^ *	6032
2D culture	~0.5–5 × 10^4^/cm^2 †^	1.45 × 10^6 ‡^	6034

* Pooled sample from two devices. ^†^ A typical value is shown as the number of cells can be easily adjusted in 2D cultures. ^‡^ Mixture of HepG2 cells, human umbilical vein endothelial cells (HUVECs), and mesenchymal stem cells (MSCs) at a ratio of 3:3:0.5.

**Table 2:** The top 10 gene ontology (GO) terms that were enriched in the tubular liver tissue.

GO ID	Description	*p* Value
GO:0018105	peptidyl-serine phosphorylation	5.01 × 10^−4^
GO:0006096	glycolytic process	5.32 × 10^−4^
GO:0097421	liver regeneration	4.01 × 10^−4^
GO:0045943	positive regulation of transcription from RNA polymerase I promoter	5.23 × 10^−3^
GO:0033617	mitochondrial respiratory chain complex IV assembly	1.34 × 10^−2^
GO:0016311	dephosphorylation	1.56 × 10^−2^
GO:0071539	protein localization to centrosome	1.68 × 10^−2^
GO:0006895	Golgi to endosome transport	1.86 × 10^−2^
GO:0070194	synaptonemal complex disassembly	2.21 × 10^−2^
GO:0022037	metencephalon development	2.21 × 10^−2^

**Table 3:** The top 10 GO terms that were enriched in 2D culture.

GO ID	Description	*p* Value
GO:0032418	lysosome localization	9.57 × 10^−4^
GO:0048041	focal adhesion assembly	3.21 × 10^−3^
GO:0001682	tRNA 5′-leader removal	7.95 × 10^−3^
GO:0016236	macroautophagy	1.51 × 10^−2^
GO:0034613	cellular protein localization	1.65 × 10^−2^
GO:0030307	positive regulation of cell growth	2.11 × 10^−2^
GO:0070584	mitochondrion morphogenesis	2.31 × 10^−2^
GO:1900186	negative regulation of clathrin-mediated endocytosis	2.49 × 10^−2^
GO:0090155	negative regulation of sphingolipid biosynthetic process	2.49 × 10^−2^
GO:0007094	mitotic spindle assembly checkpoint	2.55 × 10^−2^

## Data Availability

The data presented in this study are available on request from the corresponding author.
